# Confirmatory Factor Analysis of the Combined Social Phobia Scale and Social Interaction Anxiety Scale: Support for a Bifactor Model

**DOI:** 10.3389/fpsyg.2017.00070

**Published:** 2017-02-02

**Authors:** Rapson Gomez, Shaun D. Watson

**Affiliations:** Faculty of Health, School of Health Sciences and Psychology, Federation University AustraliaBallarat, VIC, Australia

**Keywords:** social phobia scale, social interaction anxiety scale, bifactor model, omega hierarchical, external validity

## Abstract

For the Social Phobia Scale (SPS) and the Social Interaction Anxiety Scale (SIAS) together, this study examined support for a bifactor model, and also the internal consistency reliability and external validity of the factors in this model. Participants (*N* = 526) were adults from the general community who completed the SPS and SIAS. Confirmatory factor analysis (CFA) of their ratings indicated good support for the bifactor model. For this model, the loadings for all but six items were higher on the general factor than the specific factors. The three positively worded items had negligible loadings on the general factor. The general factor explained most of the common variance in the SPS and SIAS, and demonstrated good model-based internal consistency reliability (omega hierarchical) and a strong association with fear of negative evaluation and extraversion. The practical implications of the findings for the utilization of the SPS and SIAS, and the theoretical and clinical implications for social anxiety are discussed.

## Introduction

Social anxiety refers to fear of social situations due to concerns about being judged or embarrassed, including anxiety over social interactions, with excessive levels considered to constitute a disorder, called social anxiety disorder (SAD) in the Diagnostic and Statistical Manual of Mental Disorders, 5th edition (DSM-5; American Psychiatric Association, 2013). The Social Interaction Anxiety Scale (SIAS) and the Social Phobia Scale (SPS; Mattick and Clarke, 1998) are self-report questionnaires for measuring social interaction anxiety (anxiety associated with the initiation and maintenance of social interactions) and social performance anxiety (anxiety associated with scrutiny or observation by other people while performing a task or action), respectively. The full versions of the SPS and SIAS have 20 items each. There is also a 19-item version of the SIAS. Generally, the SPS and SIAS are administered and interpreted together, with the assumption that these measures cohere to represent a global general measure of social anxiety (Safren et al., 1998). For these measures together, the current study examined support for a bifactor model, with a general factor that includes the covariance of all the SIAS and SPS items, and specific factors for the respective SIAS and SPS items.

**Figure 1:**
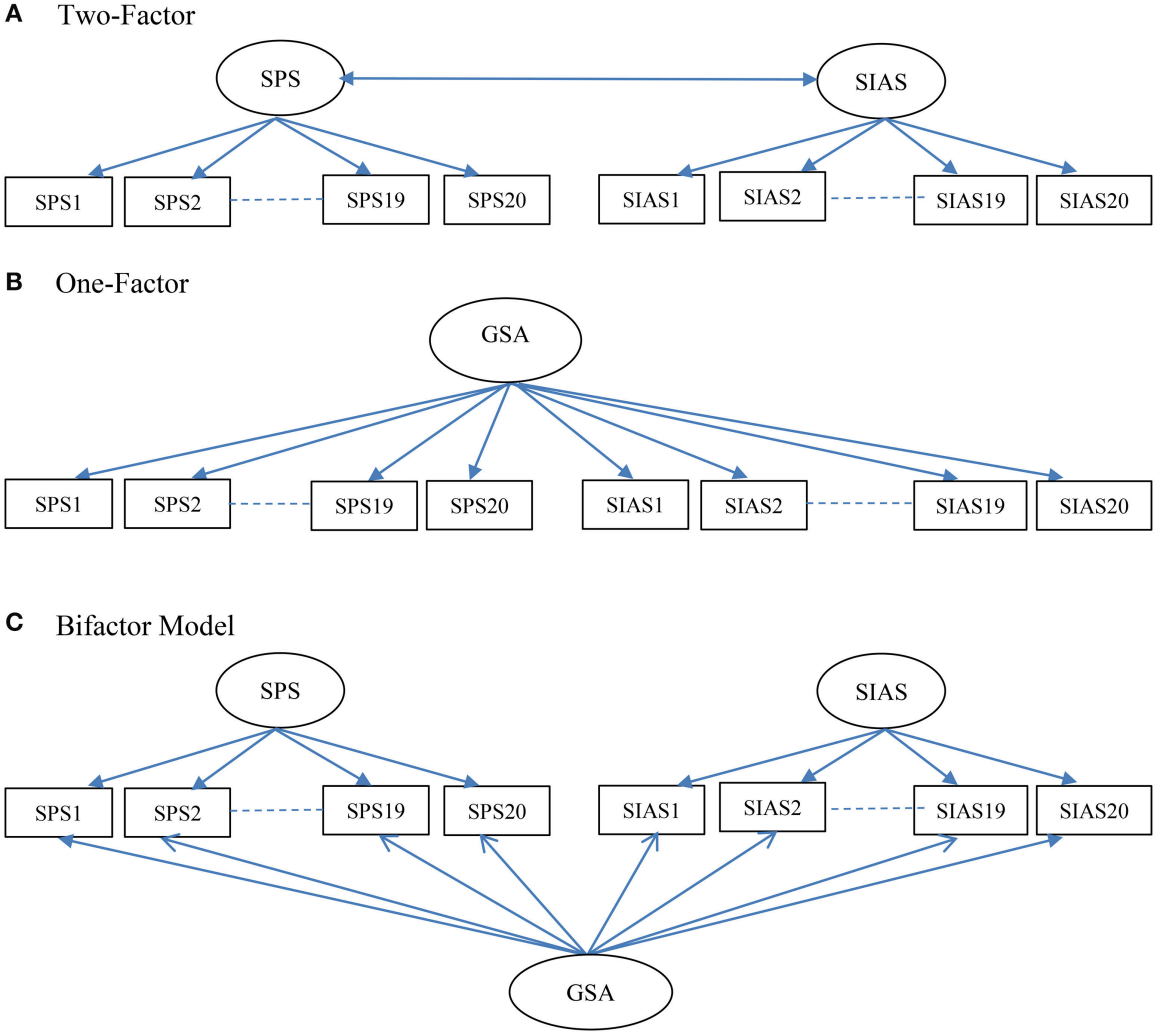
**Schematic representations of the Two-Factor (A)**, One-Factor **(B)**, and Bifactor **(C)** models of the Social Phobia Scale (SPS) and the Social Interaction Anxiety Scale (SIAS) examined in the study. GSA, general social anxiety.

When considered separately, use of total SIAS and SPS scores implies one-factor models for each of these measures. It therefore follows that when the SPS and SIAS are considered together it should reflect a two-factor model, with separate factors for the SPS and SIAS items (see Figure 1A). In this respect, it could be an oblique two-factor model as there is high correlation between the SPS and SIAS factors (Heimberg et al., 1992; Brown et al., 1997; Safren et al., 1998; Carleton et al., 2009; Heidenreich et al., 2011; Fergus et al., 2012). Despite this, principal component analysis (PCA) and exploratory factor analysis (EFA) studies with the SPS and SIAS have reported varying numbers of factor and item content for these factors. In the initial study, Mattick and Clarke (1998) examined the factor structure of the SIAS and SPS separately. They found support for a single factor for the SIAS, and three factors for the SPS (general observation anxiety; specific fears; and fear of appearing to be ill, strange, or losing control in front of other people). Other EFA factor models have also been reported. For example, Kupper and Denollet (2012) found two factors for the SPS and three factors for the SIAS. Olivares et al. (2001) found one factor for the SPS and two factors for the SIAS. Caballo et al. (2013) found three factors for the SPS (becoming nervous when being observed by other people, being self-conscious in situations where overt behaviors are expressed, and worrying about attracting attention) and three factors for the SIAS (worrying about criticism and embarrassment, easiness to interact with other people, and difficulty to interact with other people). Consistent with these findings, PCA and EFA studies of the SPS and SIAS together have shown more than the expected two factors (Habke et al., 1997; Safren et al., 1998; Heidenreich et al., 2011). For example, the joint EFA of the SPS and SIAS conducted by Safren et al. (1998) found factors for interaction anxiety, anxiety about being observed by others, and fear that others will notice anxiety symptoms.

Across community and clinical samples, multiple factors have also been supported by confirmatory factor analysis (CFA) studies. Indeed, such studies have reported better fitting models, with more than one factor when the SPS and SIAS were examined separately, and with more than two factors when they are examined together (Habke et al., 1997; Safren et al., 1998; Carleton et al., 2009; Heidenreich et al., 2011; Carter et al., 2014). For the SPS, Kupper and Denollet (2012) found most support for the three-factor structure described by Mattick and Clarke (1998), whereas Olivares et al. (2001) found most support for a one-factor model. For the SIAS, Kupper and Denollet (2012) found most support for a two-factor model, in which its 17 straightforward scored items made up one factor and the remaining three reverse scored items made up the second factor. In contrast, Olivares et al. (2001) found most support for a one-factor model for the SIAS. Rodebaugh et al. (2006; see also Rodebaugh et al., 2007) also found support for a one-factor model for the SIAS, although the reverse scored items did not contribute as well as the straightforward scored items to this factor. Independent of sample characteristics (community, general clinical, anxiety disordered, or SAD) and estimation procedures, the findings (based on at least one fit index) from CFA studies have provided mixed findings, with studies showing either good (Olivares et al., 2001; Kupper and Denollet, 2012), adequate (Heidenreich et al., 2011), or poor (Carleton et al., 2009) fit for one-factor models for the SPS and the SIAS when they were examined separately. When examined together, CFA studies have reported either good (Olivares et al., 2001; Fergus et al., 2012; Peters et al., 2012) or adequate (Heidenreich et al., 2011; Carter et al., 2014) fit for the two-factor oblique model. There are also studies that have reported poor fit (Safren et al., 1998; Carleton et al., 2009). Overall, therefore, independent of the source of the sample (clinical or community), studies that have examined the SPS and SIAS have found mixed support for the expected one-factor models when the SPS and SIAS were examined separately, and the two-factor oblique model when they were examined together.

Despite no clear support for any particular factor model, the initial and subsequent studies have used total scores for both the SIAS and SPS to examine their psychometric properties (internal consistency, test–retest reliability, and concurrent and discriminant validity). The findings from such studies have shown that both the SPS and SIAS have high internal consistency values (alpha coefficients generally in the high 0.70 and 0.80 s), test–retest reliabilities (Heimberg et al., 1992; Mattick and Clarke, 1998; Osman et al., 1998), and sound discriminant and convergent validities (Heimberg et al., 1992; Brown et al., 1997; Mattick and Clarke, 1998). For example, there is evidence that the SIAS and SPS total scores correlate positively with fear of negative evaluation (Fergus et al., 2012; Kupper and Denollet, 2012; Peters et al., 2012; Le Blanc et al., 2014). SPS and SIAS total scores have also been able to discriminate individuals with and without SAD (Heimberg et al., 1992; Brown et al., 1997; Peters, 2000; Heidenreich et al., 2011).

Overall, although there is good support for the reliabilities, discriminant and convergent validities, and clinical utility of the SPS and SIAS total scores, at best, there is only mixed support for one-factor models for the SPS and SIAS when examined separately, or the two-factor oblique model when examined jointly. Given these discrepant findings, it cannot be assumed the use of SPS and SIAS total scores is appropriate. For this practice to have credibility, better support for one-factor models for the SPS and SIAS when examined separately or jointly needs to be demonstrated.

The findings from studies of the SPS and SIAS that have supported models from one to at least three factors for each of these measures could be interpreted to mean that many of the items within the SAS and SIAS share much variance. There is empirical support for this possibility. A robust finding in past studies is the high to very high correlations between the total scores of the SPS and SIAS (Heimberg et al., 1992; Brown et al., 1997; Safren et al., 1998; Carleton et al., 2009; Heidenreich et al., 2011; Fergus et al., 2012). For example, both Fergus et al. (2012) and Heidenreich et al. (2011) reported a value of 0.84 between the SPS and SIAS latent factors. Brown et al. (1997) reported a correlation of 0.72, and Heimberg et al. (1992) found a correlation of 0.89 for observed scores. Such high correlations indicate considerable shared variance across the SPS and SIAS (Brown et al., 1997; Safren et al., 1998; Heidenreich et al., 2011), which in turn could be reflective of a common general factor. Such a factor could explain why the SIAS and SPS total scores generally have shown similar relations with many external correlates (Habke et al., 1997; Mattick and Clarke, 1998; Heidenreich et al., 2011). Consistent with a general factor, hierarchical factor analysis of the SPS and SIAS conducted by Safren et al. (1998) showed that all three of their primary factors (interaction anxiety, anxiety about being observed by others, and fear that others will notice anxiety symptoms) loaded on a single higher-order, general social anxiety factor.

There are also theoretical reasons to suspect that a factor model that includes a common general factor to cover both the SPS and SIAS is tenable. This is because there is now growing evidence that social anxiety is a single continuous dimension reflecting severity of symptoms rather than different types (e.g., Furmark et al., 2000; Stein et al., 2000; Vriends et al., 2007; El-Gabalawy et al., 2010; Ruscio, 2010). For example, Stein et al. (2000) found that symptoms of social phobia existed on a continuum of severity, with a greater number of feared situations associated with greater disability. The study found no support for social phobia subtypes based on the extent or pattern of social fears. More specific to the current study, it failed to distinguish social performance anxiety and social interaction anxiety. Furmark et al. (2000) found that three clusters of individuals with social phobia differed dimensionally along a mild–moderate–severe continuum. Ruscio (2010) found that 14 indicators of performance and interactional fears loaded on a single latent factor. Additionally, in this study, taxometric procedures provided more support for the dimensional view of social anxiety than a categorical view.

From a CFA perspective, for questionnaires with two primary factors there are at least two different ways to model a general factor: a one-factor model and a bifactor model. A higher-order factor model with two first-order factors cannot be used because with only two factors loading as indicators for the higher-order factor, this component of the model is under-identified (Brown, 2006). As applied to the SPS and SIAS together, in a one-factor model, all the items of the SPS and SIAS load onto a single general factor (shown in Figure 1B). The bifactor model (shown in Figure 1C) has three factors: a general factor for social anxiety and specific factors for the SPS and SIAS. All three factors are specified as first-order factors, with no correlations between them. This specification means that the general factor accounts for the covariance of all the SPS and SIAS items, and the SPS and SIAS factors account for the unique covariance of the SPS and SIAS items, after removing the influence of the general factor. At present there are data showing either adequate (Olivares et al., 2001; Heidenreich et al., 2011), or no (Safren et al., 1998; Carleton et al., 2009) support for the one-factor model. To date no study has examined the support for the bifactor model for the SPS and SIAS when examined jointly. There is need to examine this model as it would have important implications on how to score and interpret the scores from the SPS and SIAS.

For a bifactor model it is possible to compute the explained common variance (ECV) and the omega hierarchical (ω_*h*_; McDonald, 1999; Zinbarg et al., 2005) of the general and specific factors. In relation to the bifactor model, the ECV of a general factor is the common variance explained by the general factor divided by the total common variance, and the ECV of a specific factor is the common variance explained by the specific factor divided by the total common variance. The ECV of the general factor will be high whenever there is little common variance beyond that of the general factor. Thus, high values indicate the presence of a general factor dimension in the bifactor model (Reise et al., 2013a).

The ω_*h*_ can be interpreted as an estimator of how much variance in summed (standardized) scores can be attributed to the general factor (Brunner et al., 2012). It is obtained by dividing the amount of trait variance explained by the general factor, by the total amount of variance (trait plus error) explained by the general factor (and not the entire scale as in the case of ECV). The ω_*h*_-value for a specific factor in a bifactor model can be computed by dividing the amount of specific variance (removing the variance that is part of the general factor) explained by the factor by the total amount of variance (trait plus general plus error) explained by that factor. The values for ω_*h*_ range from 0 to 1, with 0 indicating no reliability and 1 reflecting perfect reliability. According to Reise et al. (2013a), ω_*h*_-values of at least 0.75 are preferred for meaningful interpretation of a scale. Overall, therefore, high ECV and ω_*h*_ (>0.75) values would indicate the presence of a general dimension in the bifactor model. For a first-order factor model, the comparable model-based reliability is called omega (ω; McDonald, 1999). For this model, the ω*-*value for a primary factor is computed by dividing the amount of all trait variance explained by the factor by the total amount of variance (trait plus general plus error) explained by that factor. Thus, in terms of the joint CFA analysis of the SPS and SIAS, demonstration of support for the bifactor model with high ECV and ω_*h*_ (>0.75) values would indicate the presence of a dominant social anxiety factor that would therefore justify the use of the total score from these measures.

It is to be noted that existing studies of the factor structure of the SPS and SIAS have examined both clinic-referred and community samples. Although social anxiety is commonly viewed in terms of pathology or a disorder, the examination of the factor structure of the SPS and SIAS in community samples has important relevance and implications. This is because researchers have also viewed social anxiety as a continuous trait and as noted earlier, there is now growing evidence that social anxiety is a single continuous dimension reflecting severity of symptoms rather than different types, with a greater number of feared situations associated with greater disability. Additionally, trait social anxiety has been linked to specific cognitive-affective experiences (Leary and Kowalski, 1995; Westenberg, 1998), and a number of other clinically relevant responses, such as greater heart rate reactivity and arousal (Gramer et al., 2012), higher post-interaction negative affect and attitudes about one's interaction (Shimizu et al., 2011), and poor ability to inhibit goal-irrelevant distractors thereby leading to poorer performances in highly demanding tasks (Moriya and Sugiura, 2012). Thus, the study of trait social anxiety in a general community sample has relevance. As the SIAS and SPS are two major measures of trait social anxiety (Modini et al., 2015), the examination of their psychometric properties in community samples is valuable as it could contribute to better measurement and interpretation of social anxiety scores obtained by these questionnaires. Additionally, if social anxiety is to be viewed as a single continuous dimension, as has been proposed (e.g., Furmark et al., 2000; Stein et al., 2000; Vriends et al., 2007; El-Gabalawy et al., 2010; Ruscio, 2010), and if the SIAS and SPS are to be used for facilitating clinical diagnosis of SAD, then knowing the psychometric properties of these scales in community samples where the entire spectrum of the trait underling the SAD is present would facilitate better and more reliable use of these measures.

The current study examined the fit for a bifactor model of the pooled 40 items in the SPS and SIAS in a community sample. It also compared the fit of this model with one-factor and two-factor oblique models. We did not test a higher-order factor model with two first-order primary factors because, as already pointed out, with only two factors loading as indicators for the higher-order factor, this component of the model is under-identified (Brown, 2006). Moreover, the fit of such a model would be identical to the two-factor oblique model that was also tested in the study. For the best fitting model, this study aimed to examine the external validities and reliabilities of the factors in the model. The external validities were examined in terms of the relationships of the factors with fear of negative evaluation by others [as measured by the Brief Fear of Negative Evaluation Scale, (BFNE; Leary, 1983)], and extraversion and neuroticism [as measured by the Eysenck Personality Questionnaire-Revised Short Scale (EPQ-RSS; Eysenck and Eysenck, 1991)]. Fear of negative evaluation refers to one's tendency to assume that observers are likely to evaluate one's responses and behavior critically and unfavorably. According to Reiss and McNally (1985), fear of negative evaluation can contribute to the development of anxiety. As noted earlier, fear of negative evaluation by others has shown positive associations with SIAS and SPS total scores (Fergus et al., 2012; Kupper and Denollet, 2012; Peters et al., 2012; Le Blanc et al., 2014). As for extraversion and neuroticism, there are data showing that although social anxiety is associated positively with neuroticism, and negatively with extraversion (Darvili et al., 1992; Trull and Sher, 1994; John and Srivastava, 1999), the association with extraversion is unique, and stronger with social interaction anxiety than with social performance anxiety (Naragon-Gainey and Watson, 2011) In relation to reliabilities, the aim was to compute ω_*h*_ if the bifactor model was the optimum model, or ωif a first-order model was the optimum model. In terms of findings, greater support for a bifactor model than the other models was expected. As the general factor in a bifactor model captures the variances for social anxiety in the items, and the specific factors are essentially residual factors not accounted by the general factor, it can be expected that the general factor would have stronger associations than the specific factors with external variables known to be associated with social anxiety. Thus, for this study, we expected that compared to the SPS and SIAS specific factors, the general factor will have stronger associations with fear of negative evaluation, extraversion and neuroticism, and would have more reliability than the specific factors.

## Methods

### Participants

The sample (*N* = 526) comprised of 365 females (60.5%) and 160 males (39.5%). Age ranged from 18 to 65 years (*M* = 34.03, *SD* = 11.97). Participants were recruited from the Australian states of Tasmania (*N* = 200) and Victoria (*N* = 326). These two samples were combined for the analyses of the fit of the different factor models. The mean scores (*SD*) for age in the Tasmania and Victoria samples were 26.63 (11.14) and 24.45 (9.04), respectively. The groups did not differ for age, *t*_(523)_ = 1.56, *p* = 0.120. Additional analysis indicated no differences in the relative number of male and female participants across the groups, $\chi_{\left(1\right)}^{2}$ = 0.45 (number of male and females for Tasmania were 49 and 150, respectively; and for Victoria were 150 and 236, respectively). Table 1 shows the mean (*SD*) scores for the SIAS and SPS items for the Tasmanian and Victorian samples, and the results of the independent *t*-test comparing these groups. The table also includes Cohen's *d* for these comparisons. As shown, of the 40 items compared, 12 showed significant differences. However, the *d* effect sizes for all these 12 items were either trivial or small, based on Cohen's (1992) guidelines for interpreting *d* effect sizes (<0.20 = negligible; ≥0.20 and <0.50 = small; ≥0.50 and <0.80 = medium; ≥0.80 = large). Demographic background information were also obtained from the two samples. These were different for the two subsamples. For the Tasmanian sample, 86.5% of the sample identified themselves as Caucasian, 3.5% as Indigenous Australian, 4% as Asian, 2.5% as European and 2% as others. Regarding employment status, 68.5% were employed (full-time, part-time or casual), 27% were unemployed, and the remaining participants were either on a pension or in full-time study. For the Victorian sample, 70.6% were employed (full-time, part-time or casual), 7.1% were unemployed, and the remaining participants were either on a pension or in full-time study. In terms of highest educational level completed, 73.5% were either at university or completed university studies, 11.3% completed trade studies, and the remaining completed primary or secondary education. As the Tasmanian and Victorian samples showed no difference for background characteristics, and only minimal differences for SIAS and SPS item scores, these samples can be assumed to be highly comparable.

**Table 1:** **Comparison of Tasmanian and Victorian Samples on the Social Phobia Scale (SPS) and Social Interaction Anxiety Scale (SIAS)**.

**No**.	**Brief description**	**Tasmanian**		**Victorian**		***t***	***p***	***d***
		**Mean**	***SD***	**Mean**	***SD***			
**SOCIAL PHOBIA SCALE (SPS)**								
1	Write in front of other	1.03	1.08	0.98	1.18	0.49	0.628	0.04
2	Using public toilets	0.97	1.07	0.99	1.11	−0.16	0.871	0.02
3	Others listening	1.28	1.09	1.33	1.14	−0.43	0.665	0.04
4	Staring at when walking	1.11	1.09	0.98	1.16	1.25	0.211	0.12
5	Blush when with others	0.77	1.06	0.91	1.20	−1.33	0.183	0.12
6	Entering room others	1.65	1.22	1.51	1.22	1.34	0.181	0.11
7	Shaking or trembling	0.83	1.15	0.83	1.13	−0.03	0.977	0.00
8	Sitting facing other	0.72	0.92	0.72	1.03	−0.03	0.979	0.00
9	See me faint sick or ill	0.52	0.98	0.59	0.97	−0.82	0.414	0.07
10	Drink in front of group	0.26	0.66	0.38	0.80	−1.93	0.054	0.16
11	Eat in front of stranger	0.80	1.02	0.62	1.01	1.90	0.059	0.18
12	People thinking odd	0.85	1.02	0.86	1.10	−0.08	0.937	0.01
13	Carry tray in cafeteria	0.87	1.05	0.82	1.07	0.49	0.622	0.05
14	Lose control	0.60	0.99	0.58	1.00	0.26	0.794	0.02
15	Attract attention	0.80	1.04	0.83	1.02	−0.31	0.761	0.03
16	Looked at in elevator	0.56	0.84	0.61	0.93	−0.64	0.522	0.06
17	Conspicuous	0.61	0.87	0.57	0.98	0.43	0.667	0.04
18	Speak in front of people	1.58	1.25	1.44	1.19	1.19	0.233	0.11
19	Head will shake or nod	0.21	0.61	0.40	0.83	−2.94	0.003	0.26
20	Awkward if watching	1.30	1.11	1.21	1.16	0.82	0.416	0.08
**SOCIAL INTERACTION ANXIETY SCALE (SIAS)**								
1	Speaking with authority	1.23	0.98	1.65	1.23	−4.33	< 0.001	0.38
2	Making eye contact	0.75	0.97	0.86	1.08	−1.22	0.223	0.11
3	Talk about self/feelings	1.41	1.13	1.62	1.27	−2.01	0.045	0.17
4	Mixing work people	0.67	0.91	0.90	1.04	−2.55	0.011	0.24
5	Easy making friends	1.89	1.26	1.95	1.26	−0.52	0.600	0.05
6	Meet acquaintance	0.73	0.94	0.97	1.12	−2.68	0.008	0.23
7	Mixing socially	0.90	1.06	1.16	1.15	−2.68	0.008	0.24
8	Alone with another	0.55	0.78	0.83	1.09	−3.52	< 0.001	0.30
9	Ease meeting people	1.79	1.16	2.17	1.28	−3.53	< 0.001	0.31
10	Talking with people	0.72	0.88	1.00	1.09	−3.15	0.002	0.28
11	Things to talk	1.76	1.19	2.12	1.25	−3.33	0.001	0.29
12	Expressing self	1.17	1.06	1.33	1.11	−1.69	0.092	0.15
13	Disagree with other	1.05	1.03	1.24	1.02	−2.11	0.036	0.19
14	Talking to opposite sex	0.92	1.02	1.30	1.25	−3.80	< 0.001	0.33
15	What to say in social	1.20	1.19	1.35	1.28	−1.37	0.172	0.12
16	Mixing don't know	1.43	1.10	1.60	1.27	−1.72	0.087	0.14
17	Say things embarrassing	1.05	1.08	1.22	1.21	−1.65	0.100	0.15
18	Ignored in a group	1.08	1.07	1.25	1.17	−1.80	0.072	0.15
19	Mixing in a group	1.01	0.99	1.19	1.19	−1.91	0.057	0.16
20	Greet someone	1.27	1.04	1.47	1.30	−1.93	0.054	0.17

*d, Cohen's d effect size; p, probability level; t, t-values from the t-test*.

### Material

#### Social Interaction Anxiety Scale (SIAS) and the Social Phobia Scale (SPS).

The SIAS and the SPS, both developed by Mattick and Clarke (1998), measure social interaction anxiety, and social performance anxiety, respectively. Both measures have 20-item scales, and each item is rated on a five-point Likert scale, ranging from 0 (*not at all characteristic of me)* to 4 (*extremely characteristic of me)*. While none of the SPS items require reverse scoring (all negatively worded), three items in the SIAS (item number 5, 9, and 11) require reverse scoring as they are positively worded (and all the others are negatively worded). High scores on these three items, as presented in the questionnaire, were measuring high social interaction behavior, comparable with extraversion. These three items were reverse scored prior to all analyses. Total scores range from 0 to 80 for both scales, with higher scores indicating higher levels of the social anxiety constructs. Both scales have been shown to have good reliability and validity (Heimberg et al., 1992; Mattick and Clarke, 1998). Table 2 shows the mean and standard deviation (*SD*) scores for all items in these scales for the participants in the current study. The Cronbach's alpha internal consistency values of the SPS and SIAS in the current samples were 0.94 and 0.92, respectively. The value for the combined SPS and SIAS was 0.96.

**Table 2:** **One factor model of combined Social Phobia Scale (SPS) and Social Interaction Anxiety Scale (SIAS): Completely standardized factor loadings, sources of variance**.

**No**.	**Brief description**	**Mean**	***SD***	**λ**	**Var**	***u*****^2^**
**SOCIAL PHOBIA SCALE (SPS)**						
1	Write in front of other	1.00	1.14	0.39	0.15	0.85
2	Using public toilets	0.98	1.10	0.54	0.29	0.71
3	Others listening	1.31	1.12	0.60	0.36	0.64
4	Staring at when walking	1.03	1.13	0.77	0.59	0.41
5	Blush when with others	0.86	1.15	0.64	0.41	0.59
6	Entering room others	1.56	1.22	0.75	0.56	0.44
7	Shaking or trembling	0.83	1.13	0.72	0.52	0.48
8	Sitting facing other	0.72	0.99	0.76	0.58	0.42
9	See me faint sick or ill	0.56	0.97	0.68	0.46	0.54
10	Drink in front of group	0.33	0.75	0.68	0.46	0.54
11	Eat in front of stranger	0.69	1.02	0.70	0.48	0.52
12	People thinking odd	0.86	1.07	0.80	0.63	0.37
13	Carry tray in cafeteria	0.84	1.06	0.74	0.55	0.45
14	Lose control	0.59	0.99	0.77	0.59	0.41
15	Attract attention	0.82	1.03	0.80	0.63	0.37
16	Looked at in elevator	0.59	0.89	0.74	0.55	0.45
17	Conspicuous	0.59	0.94	0.75	0.56	0.44
18	Speak in front of people	1.49	1.22	0.67	0.45	0.55
19	Head will shake or nod	0.33	0.76	0.70	0.49	0.51
20	Awkward if watching	1.24	1.14	0.80	0.64	0.36
**SOCIAL INTERACTION ANXIETY SCALE (SIAS)**						
1	Speaking with authority	1.49	1.16	0.65	0.42	0.58
2	Making eye contact	0.82	1.04	0.66	0.43	0.57
3	Talk about self/feelings	1.54	1.22	0.64	0.41	0.59
4	Mixing work people	0.81	0.99	0.70	0.49	0.51
5	Easy making friends	1.93	1.26	0.07	0.00	1.00
6	Meet acquaintance	0.88	1.06	0.73	0.53	0.47
7	Mixing socially	1.06	1.12	0.74	0.54	0.46
8	Alone with another	0.72	0.99	0.73	0.53	0.47
9	Ease meeting people	2.02	1.25	0.14	0.02	0.98
10	Talking with people	0.89	1.03	0.77	0.60	0.40
11	Things to talk	1.98	1.24	0.04	0.00	1.00
12	Expressing self	1.27	1.09	0.76	0.57	0.43
13	Disagree with other	1.17	1.03	0.50	0.25	0.75
14	Talking to opposite sex	1.16	1.18	0.64	0.41	0.59
15	What to say in social	1.29	1.24	0.84	0.70	0.30
16	Mixing don't know	1.54	1.21	0.82	0.66	0.34
17	Say things embarrassing	1.15	1.17	0.85	0.73	0.27
18	Ignored in a group	1.19	1.14	0.75	0.56	0.44
19	Mixing in a group	1.12	1.12	0.83	0.69	0.31
20	Greet someone	1.39	1.21	0.71	0.50	0.50

*λ, factor loading; Var, percentage of variance explained; u^2^, uniqueness*.

#### Brief Fear of Negative Evaluation Scale (BFNE; Leary, 1983)

The BFNE was used to measure fear of negative evaluation by others. The self-report questionnaire has 12 items, and each item is rated on a 5-point scale ranging from one (*not at all characteristic*) to five (*extremely characteristic*). An example of an item is “I am frequently afraid of other people noticing my shortcomings.” The BFNE has four reverse scored items that have been shown to be vulnerable to response bias (Rodebaugh et al., 2004; Weeks et al., 2005). Thus as proposed by others (Rodebaugh et al., 2004; Weeks et al., 2005), we computed the BFNE total scores using only the eight straightforward scored items (items 1, 3, 5, 6, 8, 9, 11, 12), referred henceforth as the BFNE-S. The BFNE-S has good internal consistency (αs > 0.92), factorial validity, and construct validity (Rodebaugh et al., 2004; Weeks et al., 2005; Carleton et al., 2011). Relevant to the current study, the study by Carleton et al. (2007) found that both the total scores of the SIAS and the SPS correlated highly with the total BFNE score (>0.60). The Cronbach's alpha internal consistency value of the BFNE-S in the current sample was 0.96.

#### Eysenck Personality Questionnaire-Revised Short Scale (EPQ-RSS; Eysenck and Eysenck, 1991)

The EPQ-RSS is a shortened version of the EPQ-R. It is a 48-item “yes/no” scale that measures three dimensions of personality: extraversion, neuroticism, and psychoticism. It also includes a lie scale to detect if respondents attempt to “fake good.” Only the extraversion and neuroticism were focused on in the current study. The EPQ-RSS has been shown to have adequate to good psychometric properties (Eysenck and Eysenck, 1991). The Cronbach's α-values for extraversion and neuroticism subscales in the current study were 0.86 and 0.83, respectively. The total scale scores for extraversion and neuroticism were used in the current study.

### Procedure

Ethics approval for the recruitment of participants in Victoria was obtained from Federation University Human Research Ethics Committee, and for participants in Tasmania from the University of Tasmania, Human Research Ethics Committee. The data for the Tasmanian and Victorian samples were collected for different student projects. While both projects included the SPS and SIAS, the measures collected for examining the external validities of the SPS and SIAS differed. Both the Victorian and Tasmanian sample were convenience samples. Pursuant to ethics approval, participants were provided with an information statement prior to their involvement informing them that completing and returning questionnaires indicated that they understood the nature of the research and freely consented to participate. The Victorian participants were recruited both directly and also on-line, via Survey Monkey. All the Tasmanian participants were recruited directly. For both sources, those recruited directly were given an envelope with questionnaires, including the SPS and SIAS, and in the case of the Tasmanian sample, the EPQ-RSS, and in the cases of the Victorian sample, the BFNE-S. Completed questionnaires were returned to research assistants (in the case of Victoria) or to a return-box left at the School of Psychology's reception counter or via post in an attached reply-paid envelope (in the case of Tasmania). All questionnaires were completed anonymously.

### Analytical procedure

All analyses used the mean and variance-adjusted weighted least squares or WLSMV, using M*plus* (Version 7) software (Muthén and Muthén, 2012). This is a robust estimator, recommended for CFA with ordered-categorical scores. This method does not assume normally distributed variables. According to measurement experts, relative to other estimators, the WLSMV estimator provides the best option for modeling categorical data, including binary scored items (Lubke and Muthén, 2004; Millsap and Yun-Tein, 2004; Beauducel and Herzberg, 2006). Brown (2006) has indicated that the estimator performs well for variables with floor or ceiling effects. Thus, the WLSMV estimator is well-suited for evaluating the ratings of the SIAS and the SPS because they involved categorical scores, and as this study involved community samples, some level of floor effect can be expected in the SIAS and the SPS ratings.

At the statistical level, the goodness-of-fit of the CFA models was examined using WLSMVχ^2^. As all types of χ^2^-values, including WLSMVχ^2^, are inflated by large sample sizes, the fit of the models was also examined using two commonly used practical fit indices: the root mean squared error of approximation (RMSEA), and the comparative fit index (CFI). The guidelines suggested by Hu and Bentler (1998) are that RMSEA-values of 0.06 or below be taken as good fit, and values >0.06 to 0.08 be considered acceptable fit. For the CFI, values of 0.95 or above are taken as indicating good model-data fit, values of 0.90 and < 0.95 are taken as acceptable fit, and values <0.90 as poor fit. It is to be noted however, that the appropriateness of these “benchmarks” has yet to be established for bifactor analyses (West et al., 2012). The WLSMVχ^2^ difference test was used to determine statistical differences between models. This study used the option available in M*plus* to compute the WLSMVχ^2^ difference values and the corresponding differences in the *df* - values. However, it has recently been argued that bifactor models have the propensity to generally fit better than first-order factor models (Morgan et al., 2015), and that the χ^2^ difference test by itself is not sufficient to ascertain the acceptability of bifactor models over other models (e.g., Rodriguez et al., 2016; Bonifay et al., 2017). They have suggested that bifactor model be also judged on substantive and conceptual grounds, and other fit indices, such as the ω_*h*_ and ECV-values of the general and specific factors (e.g., Rodriguez et al., 2016; Bonifay et al., 2017). As pointed out earlier, high ECV and ω_*h*_ (>0.75) values for the general factor would indicate the presence of a general dimension in the bifactor model. These were also considered in the current study.

The relevant internal consistency omega values were computed using the procedure illustrated by Reise et al. (2013a), and the ECV-values were computed using the procedure illustrated by Reise et al. (2013b). The external validities of the factors in the optimum model with extraversion and neuroticism were examined for only the Tasmanian sample (as the EPQ-R was not completed by the Victorian sample); and the external validities of the factors in the optimum model with BFNE-S scores was examined for only the Victorian sample as (the BFNE-S was not completed by the Tasmanian sample). The analyses for the two subsamples were conducted separately, and for both analyses, the optimum model was extended to include the observed total neuroticism and extraversion score (Tasmanian sample), or the BFNE-S scores (Victorian sample). These scores were correlated with the latent factor scores for the optimum model.

## Results

### Fit of the models tested in the study

Supplementary Tables 1–3 show the correlation matrices for the 40 SPS and SIAS items. Table 3 shows the results of all the CFA models tested^1^. Based on guidelines proposed by Hu and Bentler (1998), for the one-factor model, the RMESA-value indicated acceptable fit, whereas the CFI-value indicated poor fit. For the two-factor model, both the RMSEA and CFI-values indicted acceptable fit. For the bifactor model, both the RMSEA and CFI-values indicted good fit. Table 3 also shows that the bifactor model had better fit than the other two models tested. The correlation between the SPS and SIAS factors in the two-factor was very high at 0.81 (*p* < 0.001), thereby indicative of a general factor. Taken together, these findings are most supportive of the bifactor model.

**Table 3:** **Fit of the factor models of the combined Social Phobia Scale (SPS) and the Social Interaction Anxiety Scale (SIAS)**.

**Model (M)**	**χ^2^**	***df***	**RMSEA [90% CI]**	**CFI**	**Models Compared**		
					**ΔM**	**Δ*df***	**Δχ^2^**
One-factor (O)	3247.532^***^	740	0.080 [0.077,0.083]	0.890	–		
Two-factor (T)	2153.528^***^	739	0.060 [0.057,0.063]	0.938	O–T	1	129.396^***^
Bifactor (B)	1463.617^***^	700	0.046 [0.042,0.049]	0.967	O–B	40	861.046^***^
					T–B	39	474.252^***^

*CFI, comparative fit index; CI, confidence interval; RMSEA, root mean square error of approximation*.

*** *p < 0.001*.

### Factor loadings for the factors in the bifactor model

Table 4 presents the completely standardized factor loadings of the forty SPS and SIAS items on the general and specific factors in the bifactor model. As indicated, for the general factor, with the exception of the reverse scored SIAS items 5, 9, and 11, all the other 37 SPS and all SIAS items showed salient loadings on the general factor, based on Thurstone's (1947) classical criterion for “salience” as standardized loading ≥0.3.

**Table 4:** **Bifactor model of combined Social Phobia Scale (SPS) and Social Interaction Anxiety Scale (SIAS): Completely standardized factor loadings and sources of variance**.

**SOCIAL ANXIETY FACTORS**									
**No**.	**Brief description**	**SPS (Specific)**		**SIAS (Specific)**		**General**		***h*****^2^**	***u*****^2^**
		**λ**	**Var**	**λ**	**Var**	**λ**	**Var**		
**SOCIAL PHOBIA SCALE (SPS)**									
1	Write in front of other	0.26	0.07			0.32	0.10	0.17	0.83
2	Using public toilets	0.37	0.14			0.45	0.21	0.34	0.66
3	Others listening	0.41	0.16			0.50	0.25	0.42	0.58
4	Staring at when walking	0.46	0.21			0.67	0.44	0.65	0.35
5	Blush when with others	0.32	0.10			0.58	0.34	0.44	0.56
6	Entering room others	0.35	0.12			0.68	0.46	0.58	0.42
7	Shaking or trembling	0.43	0.18			0.62	0.39	0.57	0.43
8	Sitting facing other	0.52	0.27			0.62	0.39	0.65	0.35
9	See me faint sick or ill	0.57	0.32			0.51	0.26	0.58	0.42
10	Drink in front of group	0.56	0.31			0.52	0.27	0.57	0.43
11	Eat in front of stranger	0.43	0.19			0.59	0.35	0.54	0.46
12	People thinking odd	0.35	0.12			0.74	0.54	0.66	0.34
13	Carry tray in cafeteria	0.49	0.24			0.62	0.39	0.62	0.38
14	Lose control	0.49	0.24			0.65	0.42	0.66	0.34
15	Attract attention	0.42	0.18			0.70	0.49	0.67	0.33
16	Looked at in elevator	0.54	0.29			0.59	0.35	0.64	0.36
17	Conspicuous	0.57	0.33			0.58	0.34	0.66	0.34
18	Speak in front of people	0.24	0.06			0.64	0.41	0.46	0.54
19	Head will shake or nod	0.55	0.30			0.54	0.29	0.59	0.41
20	Awkward if watching	0.40	0.16			0.72	0.52	0.68	0.32
**SOCIAL INTERACTION ANXIETY SCALE (SIAS)**									
1	Speaking with authority			0.04	0.00	0.68	0.46	0.46	0.54
2	Making eye contact			−0.04	0.00	0.70	0.49	0.49	0.51
3	Talk about self/feelings			−0.04	0.00	0.68	0.46	0.46	0.54
4	Mixing work people			0.13	0.02	0.72	0.51	0.53	0.47
5	Easy making friends			0.53	0.28	0.02	0.00	0.28	0.72
6	Meet acquaintance			0.12	0.01	0.75	0.56	0.58	0.42
7	Mixing socially			0.27	0.07	0.73	0.54	0.61	0.39
8	Alone with another			0.09	0.01	0.76	0.57	0.58	0.42
9	Ease meeting people			0.79	0.62	0.07	0.00	0.63	0.37
10	Talking with people			0.21	0.04	0.78	0.61	0.65	0.35
11	Things to talk			0.62	0.38	−0.03	0.00	0.38	0.62
12	Expressing self			−0.02	0.00	0.79	0.63	0.63	0.37
13	Disagree with other			−0.02	0.00	0.52	0.27	0.27	0.73
14	Talking to opposite sex			0.07	0.01	0.66	0.44	0.44	0.56
15	What to say in social			0.15	0.02	0.85	0.73	0.75	0.25
16	Mixing don't know			0.21	0.04	0.82	0.67	0.72	0.28
17	Say things embarrassing			0.08	0.01	0.88	0.77	0.78	0.22
18	Ignored in a group			0.14	0.02	0.77	0.59	0.61	0.39
19	Mixing in a group			0.28	0.08	0.83	0.68	0.76	0.24
20	Greet someone			0.13	0.02	0.73	0.53	0.55	0.45
**EXPLAINED COMMON VARIANCE AND OMEGA HIERARCHICAL**									
Explained common variance		0.18		0.07		0.75			
Omega hierarchical		0.34		0.08		0.85			

*λ, factor loading; Var, percentage of variance explained; h^2^, communality; u^2^, uniqueness*.

For the SPS specific factor 18 items showed salient loadings. The non-salient items were items 1 and 18. Although 18 SPS items had salient loadings on the SPS factor, in an absolute sense, only three of these items (item 9, general = 0.51 and specific = 0.57; item 10, general = 0.52 and specific = 0.56; item 19, general = 0.54 and specific = 0.55) had (slightly) higher loadings on the specific factor than the general factor. For the SIAS specific factor, only the positively word items 5, 9, and 11 showed salient loadings. Their loadings for the specific factor were 0.53, 0.79, and 0.62, respectively; and the loadings for the general factor were 0.02, 0.07 and −0.03, respectively. To enable a better understanding of the low loadings for the positively word items on the general factor, the loadings of these items on the one-factor model was examined. These loadings are presented in Table 2. As shown, the loading for items 5, 9, and 11 were 0.07, 0.14, and 0.04, respectively, thereby suggesting that these items comprised mainly of variances that can be attributed to uniqueness and/or error.

### Explained Common Variance (ECV) and internal consistency reliability of the factors in the bifactor model

Table 4 includes the ECV, and ω_*h*_ of the general and specific factors. As shown in Table 4, the ECV for the general factor was 0.75, and the ECV-values for the SPS and SIAS specific factors were 0.18 and 0.07, respectively. Most of the variance for the SIAS specific factor came from the three revered scores items. The ω_*h*_-value for the general factor was 0.85, and the values for SPS and SIAS specific factors were 0.34 and 0.08, respectively.

### External validities of the factors of the bifactor model

Table 5 shows the correlations of BFNE-S and EPQ-RSS scores with the factors in the bifactor model. As shown, the model for the Victorian sample in which BFNE-S scores were correlated with the factors of the bifactor model indicated significant and positive correlations for BFNE-S observed scores with the general factor (*r* = 0.70, *p* < 0.001) and the SPS specific factor (*r* = 0.12, *p* < 0.01). BFNE-S observed scores was not associated with the SIAS specific factor (*r* = 0.16, *ns*). The model for the Tasmanian sample in which extraversion and neuroticism were correlated with the factors of the bifactor model indicated that for extraversion, there was significant and negative correlations with the general factor (*r* = −0.42, *p* < 0.001) and with the SIAS specific factor (*r* = −0.64, *p* < 0.001), and no significant association with SPS specific factor (*r* = −0.16, *ns*). For neuroticism, there was significant and positive correlation with only the general factor (*r* = 0.15, *p* < 0.05), and no associations with SIAS (*r* = 0.14, *ns*) and SPS (*r* = 0.01, *ns*) specific factors.

**Table 5:** **Correlations of BFNE-S and EPQ-RSS scores with the factors in the bifactor model**.

	**General**	**SPS (Specific)**	**SIAS (Specific)**
**BRIEF FEAR OF NEGATIVE EVALUATION SCALE (VICTORIAN SAMPLE)**			
Total score	0.70^***^	0.12^**^	0.16
**EYSENCK PERSONALITY QUESTIONNAIRE-REVISED SHORT SCALE (TASMANIAN SAMPLE)**			
Extraversion	−0.42^***^	−0.16	−0.64^***^
Neuroticism	0.15^*^	0.01	0.14

*SIAS, Social Interaction Anxiety Scale; SPS, Social Phobia Scale*.

*** p < 0.001;

** p < 0.01;

* *p < 0.05*.

For the BFNE-S, the effect size for the association involving the general factor was large, and the effect size for the association involving the SPS specific factor was small, based on the guidelines proposed by Hemphill (2003) that *r* < 0.2 = small, 0.2–0.3 = medium or moderate, and >0.30 = large. For extraversion, the effect size for the association involving both the general factor and the SIAS specific factor were large, and for neuroticism, the effect size for the association with the general factor was small.

## Discussion

The study examined and compared one-factor, two-factor, and bifactor models of the pooled SPS and SIAS items. The one-factor model had mixed fit, with the RMESA-value indicating acceptable fit, and the CFI-value indicating poor fit. For the two-factor model, there was acceptable fit in terms of both RMSEA and CFI-values. The bifactor model showed good fit in terms of both the RMSEA and CFI-values. The acceptable support for the two-factor is consistent with existing data (Olivares et al., 2001; Fergus et al., 2012; Peters et al., 2012), as is mixed support for the one-factor model (Olivares et al., 2001; Heidenreich et al., 2011). The chi-square difference test indicated that the bifactor model had better fit than the other two models, and the two-factor model had better fit than the one-factor model. Overall, therefore, although there was reasonably good fit for the two-factor model, the bifactor model was the better structural model to represent the combined ratings on the SPS and SIAS items. This was as expected. As this is the first study to directly examine a bifactor model for the pooled SPS and SIAS items, this finding is new.

The findings for the bifactor model showed that with the exception of the three reverse scored SIAS items (5, 9, and 11), all the other 37 straightforward scored items in the SIAS and SPS showed salient loadings on the general factor. For the SIAS specific factor, only the three reverse scored items showed salient loadings. Although 18 SPS items had salient loadings on the SPS factor, in an absolute sense, only three items (items 9, 10, and 19) had higher loadings on the specific factor than the general factor. These findings indicate that the general factor is dominant over the SPS and SIAS specific factors.

For the bifactor model, the ECV of the general factor was 0.75, and the ECV of the specific factors for SPS and SIAS were 0.18 and 0.07, respectively. Thus, the general factor accounted for around three times more common variance than the two specific factors together. In relation to internal consistency reliability values, the findings showed that the ω_*h*_ for the general factor was 0.85, and the ω_*h*_ for the SPS and SIAS specific factors were 0.34 and 0.08, respectively. According to Reise et al. (2013a), ω_*h*_-values of at least 0.75 are preferred for meaningful interpretation of a scale. Taken together, these findings indicate that only the general factor has sufficient variance and reliability for meaningful interpretation.

Reise et al. (2013b) have recommended that for a bifactor model, ECV-values >0.60, and ω_*h*_-values >0.70 for the general factor be used to determine whether the general factor shows sufficient unidimensionality so that scores obtained by summing all the items are not biased. As the ECV and ω_*h*_-values for the bifactor model for the SPS and SIAS were 0.75 and 0.81, respectively, it can be assumed that the general factor has sufficient unidimensionality. This also means that use of the total score, based on all the items of the SPS and SIAS, will not be biased.

The findings for the bifactor model also showed that the general factor was associated positively with large effect size with fear of negative evaluation. This finding was expected as there is existing data showing high correlations for BFNE total score with the total scores of the SIAS and the SPS (Carleton et al., 2007). Although the SPS specific factor was associated with fear of negative evaluation, the effect size was small. The general factor showed a significant and negative correlation with high effect size with extraversion, whereas it correlated significantly, positively and with low effect size with neuroticism. Also, only the SIAS specific factor showed significant correlation with extraversion. This correlation was significant and negative, and of large effect size. This finding is not surprising as the variances for the SIAS specific factor came mostly from the three reversed worded items that, as noted earlier, reflected high social interaction, comparable with extraversion. None of the other correlations for extraversion or neuroticism with the specific factors were significant. Taken together, these findings indicate different magnitude and directions of relations between the general and specific factors with fear of negative evaluation, extraversion, and neuroticism, thereby supporting the external validity of the general factor and weaker support for the specific factors. However, the findings involving the specific factors need to be viewed with caution as the specific factors had very low reliabilities and common variances, which limit a meaningful interpretation of findings involving these factors.

Overall, when the findings in the study are considered together, it can be concluded that while there is support for the bifactor model, only the general factor can be meaningfully interpreted, and the scores of the items in this factor provide an unbiased measure for the ratings on the SPS and SIAS when they are used together. It worth noting, however, that while the general factor explains most of the covariance in the scores of the SAS and SIAS, the findings in the current study showed that this is especially so for the 37 straightforward scored SIAS and SPS items.

The findings in the study have implications for the use of the SAS and SIAS. The findings indicating no support for the specific factors for SPS and SIAS mean that when the SPS and SIAS are used concurrently they do not provide independent measures of social performance anxiety or social interaction anxiety. Thus, they should not be used for measuring social performance anxiety and social interaction anxiety, or other subtypes of social anxiety that have been suggested by the results of several past factor analyses studies (Habke et al., 1997; Safren et al., 1998; Carleton et al., 2009; Heidenreich et al., 2011; Carter et al., 2014). It is to be noted that the lack of support found for independence of the SPS and SIAS is consistent with other studies (Furmark et al., 2000; Stein et al., 2000; Ruscio et al., 2008).

The support for the general factor indicates that when the SPS and SIAS are used concurrently, the most prudent way of scoring them is to sum the ratings on the scales together to obtain an overall score of general social anxiety. This has not been proposed so far. At present, when the SPS and SIAS are used concurrently, separate scores for social performance anxiety and social interaction anxiety are computed. In relation to our recommendation to use the summed score, as the findings here showed that the three reverse scored SIAS items had low non-salient loadings on the general factor, it can be argued that that the total score is better derived from the sum of the 20 SPS items and the 17 straightforward scored SIAS items. The exclusion of the three reverse scored items for scoring the SIAS is consistent with exiting recommendations (Rodebaugh et al., 2006, 2007).

The findings here also have theoretical and clinical implications for social anxiety. First, the support for a dominant general anxiety factor is consistent with growing evidence that social anxiety is better viewed as a single continuous dimension, reflecting low to high severity of social anxiety symptoms, rather than different types (e.g., Furmark et al., 2000; Stein et al., 2000; Vriends et al., 2007; Ruscio, 2010; El-Gabalawy et al., 2010). A single continuous dimension reflecting low to high severity of social anxiety symptoms also means that the diagnosis of SAD can be made along a continuum of severity, rather than in dichotomous terms related to either presence or absence of SAD. This is notable, as existing data indicate that dimensional scores are far more predictive of a SAD diagnosis than categorical scores (Ruscio, 2010). Using a dimensional approach will also enable clinicians to track ongoing changes in the level of social anxiety following treatment.

In concluding, the findings in the current study add in important ways to the literature on the SPS and the SIAS, especially how these measures are to be scored for research and clinical practice. However, the findings and interpretations made in this study have to be viewed with limitations in mind. First, there was no information on those who did not respond to the invitation to participate. Thus, it is not known how the missing data from these individuals may have impacted our findings. Second, because participants were from the general community, the findings here could be biased, and not applicable to other samples, including clinical samples and those with a diagnosis of SAD. However, as already noted in the introduction, social anxiety has also been viewed as a continuous trait linked to specific cognitive-affective, physiological, attitudinal, and attention performance processes. For this reason, and if social anxiety is a single continuous dimension, the possibility of which we have raised, knowing the psychometric properties of the SIAS and SPS in community samples would be valuable as it could contribute to better interpretation of social anxiety scores obtained by these questionnaires. Related to our sample limitation, we also used a convenience sample. Third, it is possible that demographic factors such as age, sex, and ethnicity could influence ratings on the SPS and SIAS. The failure to control for these effects in the study could have confounded the results. Related to this is that the sample comprised three times more females than males. It is to be noted however, that previous studies have not found significant sex differences for the SPS and the SIAS (Olivares et al., 2001; Caballo et al., 2013). Fourth, the findings reported here are based on a single study. Fifth, as the ratio of participants for every estimated parameter for the most complex model (bifactor model) was low (4.4:1), the number of participants in the study could be considered low for stable estimates. However, some researchers have suggested that this number of participants is satisfactory for CFA (Brown, 2006). As a consequence, there is a need for validating of the findings before the findings can be generalized. It is suggested that more studies be conducted in this area, taking into consideration the limitations highlighted here.

## Ethics statement

Federation University Human Research Ethics Committee, and the University of Tasmania, Human Research Ethics Committee. Prior to completing the questionnaires participants were required to read through a plain language information statement about the study, informing them of details of the study pursuant to the requirements of the ethics committee approval (e.g., right to withdraw, anonymity, etc.). Consent to participate was implied through completion of the questionnaires, and the information statement informed participants that submitting and/or returning questionnaires indicates that they understood the nature of the research and freely consented to participate.

## Author contributions

RG and SW: Conception and design of research; analysis and interpretation of data; drafting and revising manuscript; approval of final manuscript; accountability for accuracy; and integrity of work.

### Conflict of interest statement

The authors declare that the research was conducted in the absence of any commercial or financial relationships that could be construed as a potential conflict of interest.

## Footnotes

^1^There were no missing values for the data set analyzed in the current study.
