# Supplementary material for: Confirmatory Factor Analysis of the Combined Social Phobia Scale and Social Interaction Anxiety Scale: Support for a Bifactor Model
Source: Front Psychol. 2017 Feb 2;8:70. doi: 10.3389/fpsyg.2017.00070 (PMC5288358; doi:10.3389/fpsyg.2017.00070)
Supplement: Supplementary file 1 [file Table1.PDF]

Supplementary Table 1

Correlations between Social Interaction Anxiety Scale Items

	SIA 1	SIA 2	SIA 3	SIA 4	SIA 5	SIA 6	SIA 7	SIA 8	SIA 9	SIA 10	SIA 11	SIA 12	SIA 13	SIA 14	SIA 15	SIA 16	SIA 17	SIA 18	SIA 19	SIA 20
SIA 1	-																			
SIA 2	.342	-																		
SIA 3	.343	.530	-																	
SIA 4	.321	.513	.557	-																
SIA 5	.297	.354	.497	.600	-															
SIA 6	.258	.431	.515	.693	.607	-														
SIA 7	.320	.426	.516	.592	.524	.610	-													
SIA 8	.350	.531	.488	.644	.461	.623	.633	-												
SIA 9	.366	.370	.472	.518	.430	.443	.582	.627	-											
SIA 10	.394	.423	.471	.503	.411	.453	.546	.603	.675	-										
SIA 11	.265	.472	.442	.605	.400	.515	.429	.594	.572	.660	-									
SIA 12	.310	.401	.511	.624	.534	.574	.591	.600	.588	.545	.643	-								
SIA 13	.279	.441	.472	.661	.430	.680	.586	.645	.553	.582	.633	.645	-							
SIA 14	.310	.342	.528	.609	.503	.493	.606	.596	.668	.583	.567	.705	.646	-						
SIA 15	.280	.437	.459	.609	.498	.609	.541	.632	.584	.593	.596	.683	.676	.747	-					
SIA 16	.278	.427	.460	.635	.533	.516	.594	.676	.590	.592	.555	.588	.601	.638	.677	-				
SIA 17	.235	.427	.500	.652	.457	.551	.557	.695	.585	.549	.545	.620	.605	.697	.661	.746	-			
SIA 18	.276	.337	.429	.519	.527	.584	.529	.457	.407	.399	.449	.542	.492	.426	.538	.428	.470	-		
SIA 19	.353	.443	.453	.522	.519	.400	.667	.617	.656	.661	.497	.601	.532	.660	.533	.580	.621	.462	-	
SIA 20	.360	.452	.437	.724	.525	.656	.624	.616	.520	.531	.582	.662	.637	.597	.700	.649	.660	.685	.609	-

Note. SIA = Social Interaction Anxiety
