# Supplementary material for: Confirmatory Factor Analysis of the Combined Social Phobia Scale and Social Interaction Anxiety Scale: Support for a Bifactor Model
Source: Front Psychol. 2017 Feb 2;8:70. doi: 10.3389/fpsyg.2017.00070 (PMC5288358; doi:10.3389/fpsyg.2017.00070)
Supplement: Supplementary file 2 [file Table2.PDF]

Supplementary Table 2

Correlations between Social Interaction Anxiety Scale Items and Social Phobia Scale Items

	SIA 1	SIA 2	SIA 3	SIA 4	SIA 5	SIA 6	SIA 7	SIA 8	SIA 9	SIA 10	SIA 11	SIA 12	SIA 13	SIA 14	SIA 15	SIA 16	SIA 17	SIA 18	SIA 19	SIA 20
SP 1	.289	.364	.348	.441	.444	.521	.386	.382	.347	.310	.404	.466	.401	.351	.421	.412	.338	.479	.345	.573
SP 2	.257	.338	.329	.514	.417	.444	.465	.529	.405	.396	.501	.531	.471	.477	.498	.436	.439	.426	.454	.507
SP 3	.243	.356	.372	.462	.441	.506	.454	.428	.335	.324	.362	.441	.422	.425	.497	.397	.395	.495	.314	.495
SP 4	.284	.365	.355	.433	.332	.417	.395	.461	.403	.391	.429	.524	.404	.482	.530	.396	.445	.399	.473	.504
SP 5	.035	.021	-.046	.066	.049	.039	.015	.061	.088	.019	.094	-.001	.000	-.016	.037	.055	.044	.036	-.059	.003
SP 6	.224	.406	.378	.484	.387	.475	.542	.478	.397	.366	.452	.547	.471	.494	.527	.495	.490	.403	.402	.506
SP 7	.158	.303	.322	.418	.349	.445	.425	.404	.356	.398	.436	.512	.426	.482	.530	.415	.389	.405	.314	.465
SP 8	.235	.416	.408	.529	.393	.482	.512	.568	.452	.423	.471	.545	.516	.510	.562	.538	.473	.414	.498	.573
SP 9	.002	.076	-.034	.064	.097	.045	.024	.065	.042	.109	.111	.076	.064	.043	.093	.105	.024	.066	.053	.085
SP 10	.210	.395	.388	.476	.456	.526	.476	.493	.345	.396	.429	.568	.397	.462	.546	.440	.409	.493	.404	.539
SP 11	-.031	-.003	-.007	.000	.032	.030	.015	.014	-.030	.017	.061	.013	-.001	-.011	.002	.017	-.029	-.006	-.021	.018
SP 12	.222	.304	.468	.533	.527	.490	.578	.529	.444	.443	.465	.604	.476	.571	.547	.515	.486	.496	.464	.552
SP 13	.182	.178	.269	.311	.356	.370	.323	.311	.305	.378	.333	.371	.345	.370	.444	.296	.337	.344	.346	.387
SP 14	.233	.294	.314	.384	.444	.439	.376	.355	.351	.369	.389	.511	.413	.429	.470	.421	.381	.457	.381	.436
SP 15	.302	.350	.430	.547	.512	.565	.506	.509	.428	.409	.498	.659	.528	.578	.576	.484	.474	.530	.454	.580
SP 16	.208	.357	.363	.519	.403	.564	.445	.491	.384	.376	.466	.567	.485	.491	.565	.449	.475	.534	.332	.573
SP 17	.325	.375	.454	.610	.540	.580	.549	.558	.482	.477	.532	.686	.531	.585	.630	.523	.493	.578	.475	.624
SP 18	.270	.337	.387	.558	.401	.527	.461	.419	.417	.443	.421	.583	.501	.523	.529	.430	.463	.460	.456	.541
SP 19	.231	.313	.333	.527	.390	.546	.479	.504	.347	.443	.490	.573	.495	.496	.532	.467	.489	.517	.377	.571
SP 20	.249	.302	.350	.468	.352	.511	.455	.475	.397	.384	.433	.544	.485	.482	.489	.407	.421	.410	.402	.516

Note. SIA = Social Interaction Anxiety; SP = Social Phobia
