# Supplementary material for: Confirmatory Factor Analysis of the Combined Social Phobia Scale and Social Interaction Anxiety Scale: Support for a Bifactor Model
Source: Front Psychol. 2017 Feb 2;8:70. doi: 10.3389/fpsyg.2017.00070 (PMC5288358; doi:10.3389/fpsyg.2017.00070)
Supplement: Supplementary file 3 [file Table3.PDF]

Supplementary Table 3

Correlations between Social Phobia Scale Items

	SP 1	SP 2	SP 3	SP 4	SP 5	SP 6	SP 7	SP 8	SP 9	SP 10	SP 11	SP 12	SP 13	SP 14	SP 15	SP 16	SP 17	SP 18	SP 19	SP 20
SP 1	-																			
SP 2	.458	-																		
SP 3	.516	.519	-																	
SP 4	.468	.578	.450	-																
SP 5	.052	.016	.018	.011	-															
SP 6	.511	.573	.453	.615	.037	-														
SP 7	.448	.447	.465	.628	.081	.647	-													
SP 8	.491	.503	.525	.501	.108	.615	.593	-												
SP 9	.060	.064	.039	.135	.444	.175	.193	.131	-											
SP 10	.542	.528	.529	.639	.105	.619	.708	.633	.165	-										
SP 11	.098	-.023	-.001	.014	.364	.006	.026	.054	.534	.067	-									
SP 12	.562	.490	.553	.561	.032	.581	.551	.547	.051	.641	.039	-								
SP 13	.389	.207	.365	.342	.033	.363	.370	.292	.023	.400	.008	.464	-							
SP 14	.468	.441	.444	.398	.052	.495	.505	.473	.103	.529	.018	.525	.367	-						
SP 15	.558	.542	.545	.611	.049	.618	.643	.607	.186	.692	.040	.696	.411	.642	-					
SP 16	.560	.527	.579	.601	.122	.588	.653	.578	.155	.672	.039	.589	.374	.571	.790	-				
SP 17	.594	.527	.563	.600	.072	.606	.597	.636	.116	.662	.064	.695	.475	.589	.780	.779	-			
SP 18	.514	.460	.396	.548	.019	.560	.605	.575	.141	.619	.015	.581	.428	.477	.673	.651	.711	-		
SP 19	.525	.552	.511	.662	.114	.637	.771	.631	.159	.725	.034	.600	.434	.529	.736	.780	.737	.743	-	
SP 20	.491	.489	.464	.542	.058	.627	.555	.571	.149	.538	.052	.538	.325	.544	.640	.632	.667	.600	.651	-

Note. SP = Social Phobia
